# Development and validation of the diabetic self-management scale based on information-motivation-behavioral skills theory

**DOI:** 10.3389/fpubh.2023.1109158

**Published:** 2023-02-24

**Authors:** Zhenwei Dai, Shu Jing, Xiaoyang Liu, Haoran Zhang, Yijin Wu, Hao Wang, Weijun Xiao, Yiman Huang, Jiaqi Fu, Xu Chen, Lei Gao, Xiaoyou Su

**Affiliations:** ^1^School of Population Medicine and Public Health, Chinese Academy of Medical Sciences and Peking Union Medical College, Beijing, China; ^2^NHC Key Laboratory of Systems Biology of Pathogens, Institute of Pathogen Biology, and Center for Tuberculosis Research, Chinese Academy of Medical Sciences & Peking Union Medical College, Beijing, China

**Keywords:** type 2 diabetes mellitus, self-management, psychometric properties, validation, Information-Motivation-Behavioral Skills Model (IMB)

## Abstract

**Background:**

Self-management is important for the blood sugar control of middle-aged and elderly Type 2 diabetes mellitus (T2DM) patients, of which diet, exercise, and drug compliance are the most common components. The Information-Motivation-Behavioral Skills Model (IMB) has been widely used in health behavior management and intervention.

**Objective:**

The purpose of this study is to develop and validate the Diabetic Self-Management Scale (DSMS) based on the IMB model.

**Methods:**

Self-report survey data was collected from middle-aged and elderly T2DM patients in Zhongmu City, Henan Province, China in November 2021 using convenience sampling. The original DSMS was developed through a literature review and summary of previous similar scales using an inductive approach. Item modification was finished by a panel of specialists. Exploratory factor analysis and confirmatory factor analysis were used to evaluate the reliability, convergent validity, discriminant validity, and criterion validity of DSMS.

**Results:**

Four hundred and sixty nine T2DM patients completed the questionnaire survey. The final DSMS consists of 22 items with three dimensions, including information (five items), motivation (eight items), and behavior skills (nine items). The results of simple factor analysis showed that the KMO value was 0.839, Bartlett spherical test 2 = 3254.872, *P* < 0.001. The results of confirmatory factor analysis showed that 2/df = 2.261, RMSEA = 0.073, CFI = 0.937, TLI = 0.930, and SRMR = 0.096. The standardized factor loadings of 22 DSMS items were all above 0.6, and the CR values of 3 dimensions were all higher than 0.9. In addition, DSMS also showed good discriminant and criterion validity.

**Conclusion:**

The 22-item DSMS has good reliability and validity, and can be used to make diabetic self-management assessment regarding diet, physical activity, and medication among middle-aged and elderly Chinese T2DM patients. DSMS is of moderate length and easy to understand. It can be promoted in China in the future to understand the self-management status of middle-aged and elderly T2DM patients in China.

## 1. Introduction

With the increasing rates of aging and obesity worldwide, diabetes has become one of the most serious and common chronic diseases currently (1). According to International Diabetes Federation (IDF), there were about 537 million people suffering from diabetes in 2021 worldwide, with a global prevalence estimated to be more than 10%, and the number is expected to reach 783 million by 2045 (2). Due to the large aging population, China has now become the country with the highest prevalence of diabetes in the world and has the largest number of diabetic patients (3). According to the 2021 Global Diabetes Atlas released by International Diabetes Federation (IDF), the total number of diabetic patients in mainland China was estimated to be 140.9 million in 2021 (2). The incidence of diabetes continues to increase with age, especially after the age of 50, and among the middle-aged and elderly populations in China, the prevalence of diabetes and pre-diabetes was more than 10 and 40% respectively in 2018 (4–6). Worse still, the high incidence of diabetes in middle-and low-income countries has brought huge costs and burdens to the global health economy (7). Empirical data showed that in 2021, the direct health expenditure caused by diabetes has reached nearly 1 trillion dollars, which has increased about 316% over the past 15 years globally (2). Moreover, the socio-economic inequities in diabetes are also not conducive to the prevention and control of diabetes worldwide, especially in developing countries (8). Type 2 diabetes mellitus (T2DM) is the most common type of diabetes mellitus, accounting for 90% of all diabetic patients (9). T2DM patients may develop microvascular and macrovascular complications such as cardiovascular disease, diabetes nephropathy, and diabetes ophthalmopathy without effective control of blood sugar (10). In addition, T2DM patients have a 15% increased risk of all-cause death compared with healthy people (11). This will lead to both the compromise of life quality of T2DM patients and huge financial burden to their families (12).

The main contributing factors for T2DM were obesity and unhealthy lifestyles like sedentariness (13). Early control of T2DM and patient-centered self-management can reduce blood glucose levels and minimize complications (9). A series of randomized controlled trials indicated that lifestyle interventions, such as increasing physical activity and having a healthy diet, are simple and effective ways to control the progression of T2DM (14–18). Apart from the lifestyle changes recommended by their family, friends or doctors, most of the T2DM patients are benefiting from the diabetes medication, typically given to the control of the blood glucose and further occurrence of complications of T2DM. Hence, adhering to a doctor's prescription for hypoglycemic medications and suggestions is crucial for managing the condition and preventing the emergence of T2DM complications (4, 19, 20). However, the self-management practice of T2DM patients need further optimization and refinement, since it involves multiple aspects, such as eating habits, physical activities, and medication adherence, and might be complicated for T2DM patients to follow it strictly (21). Recent studies have found that most T2DM patients only adhere to their treatment to a moderate degree (22–24). In this case, an instrument to systematically assess eating habits, physical activities, and medication adherence is necessary for T2DM patients to evaluate their capacity for self-management. Currently, most self-management scales employed among Chinese T2DM patients were introduced from other countries, such as the Diabetes Self-Care Scale (DSCS) which evaluates the self-management ability from the perspective of diet, blood sugar detection, feet care, physical activity, and medication; Summary of Diabetes Self-Care Activities Scale (SDSCA) that contains the assessment of diet, physical activity, blood sugar detection, feet care, and smoking; The Personal Diabetes Questionnaire (PDQ) that was developed under the structure of knowledge, self-decision, self-management, and psychology; and Diabetes Care Profiles (DCP) that evaluate the mental and social health of diabetic patients from multiple dimensions (25–28). However, few studies focused on the development and validation of a self-management scale for T2DM patients in China considering the culture of the Chinese context. Additionally, the existing self-management evaluation tools for diabetes often involve multiple dimensions such as diet, physical activity, blood glucose monitoring, feet care, and drug compliance. Among them, diet, physical activity and drug treatment are of utmost concerns by majority of the diabetes and prediabetes patients, and it is also the focus of medical staff (6). Therefore, developing a tool focusing on the above mentioned three aspects to evaluate the self-management status of diabetes patients might be expected to provide a measurement applicable to a broader T2DM population.

**Figure 1:**
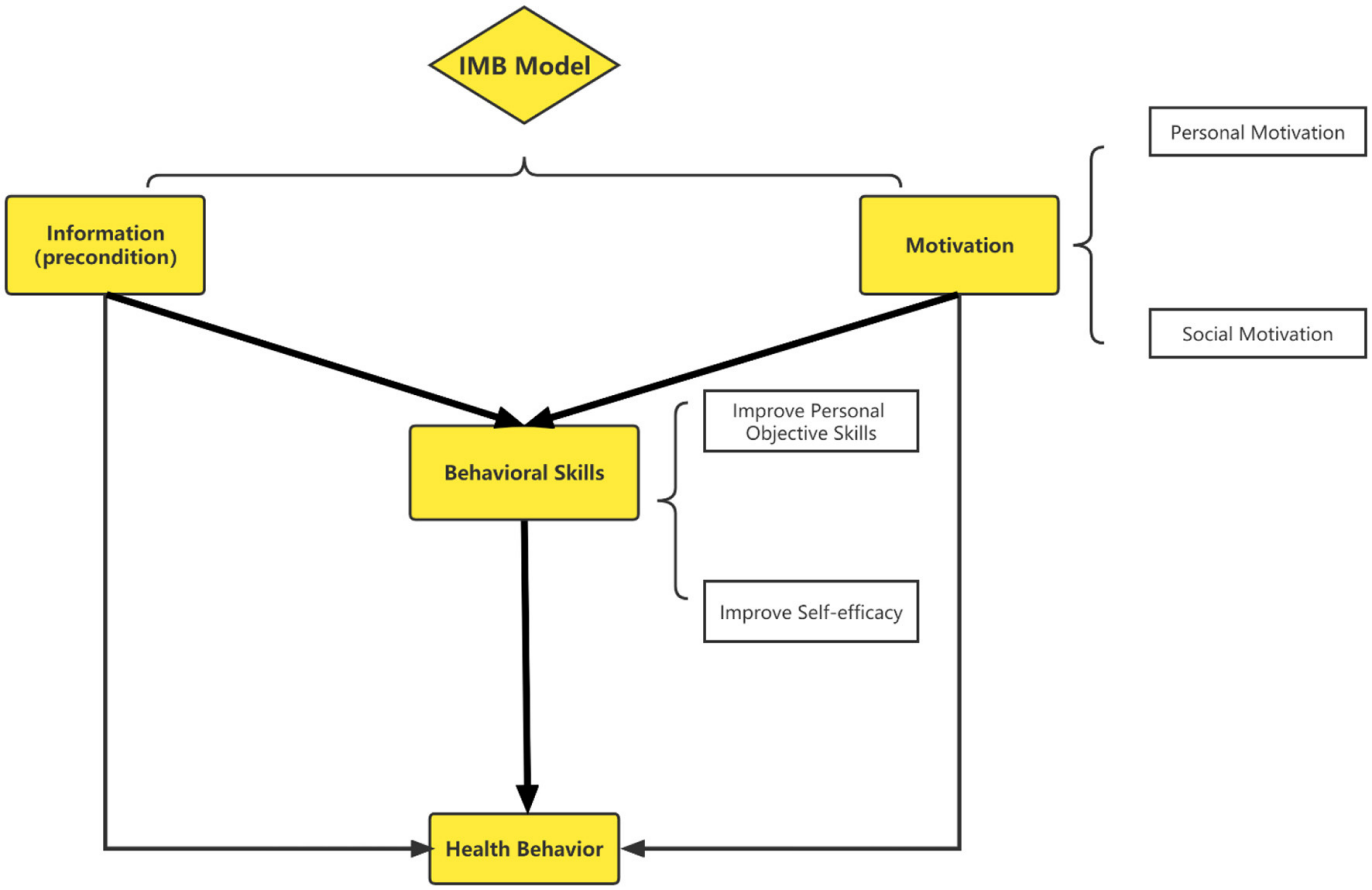
Conceptual framework of the role of IMB model on health behavior.

The Information-Motivation-Behavioral Skills Model (IMB) developed by Fisher et al. in the 1990's was originally used to evaluate the risk of HIV infection and promote the prevention of HIV/AIDS (29). According to the IMB theory, the performance of behavior requires behavior-specific information, motivation, and behavior skills. Individuals with higher levels of information, motivation and behavioral skills are more likely to adopt healthy behaviors. Therefore, measuring the level of the above three dimensions can well-predict and reflect individual's behavior. Information is a factor directly related to health-related behavior, and motivation is an additional determinant of health-related behavior. Adequate information and motivation can promote individuals to develop appropriate behavioral skills and ultimately lead to health-promoting behaviors (Figure 1) (30, 31). At present, the IMB model has demonstrated a satisfactory predictive ability to improve the compliance of self-management behavior and ameliorate the health outcome of T2DM patients, showing good practicability and maturity (32). For example, Qin compiled the IMB-SMBG questionnaire based on the IMB model to investigate the self-monitoring of blood glucose in adult type I diabetes patients (33). However, the diabetic self-management scales employed in these studies were mostly self-designed without strict validation, and few studies focused on middle-aged and elderly T2DM patients in China (34). Therefore, the purpose of the current study is to develop a diabetic self-management scale (DSMS) in Chinese middle-aged and elderly T2DM patients based on IMB theory. And it is intended to evaluate the self-management of T2DM patients from the perspective of diet, physical activity, and medication, respectively, and to provide a tool for systematically understanding of their relevant health-related behaviors.

## 2. Materials and methods

### 2.1. Scale development

The Diabetic Self-Management Scale (DSMS) developed in this study was based on the three dimensions of IMB theory, namely information, motivation, and behavioral skills. And each dimension covered items on physical activity, diet, and medication of T2DM. The information dimension was developed based on the items from the diabetes knowledge questionnaires of the Diabetes Education Project in China of Project Hope, while items of motivation and behavioral skill dimensions were developed based on comprehensive literature reading and other scales that our research team had previously developed and employed based on IMB or similar models (35, 36). Initially, a 55-item pool (15 items of information, 20 items of motivation, and 20 items of behavioral skill) was generated *via* literature review and group discussion. Then, similar items were collapsed into 1 item to avoid redundancy. For example, “Appropriate physical activities can lower my blood sugar level,” “Physical activities can make my blood sugar well controlled”, and “Blood sugar level is difficult to control if I do not exercise.” were collapsed into “Appropriate physical activities can lower my blood sugar level.” Meanwhile, some items were also removed by considering the practicability and applicability in the study population by investigators and field workers who were familiar with the study population and the local culture of the study site, such as “Do you know what your ideal weight is?.” After this phase, the 55-item pool was reduced to 43 items (13 items of information, 15 items of motivation, and 15 items of behavioral skill). Later, a panel of specialists in epidemiology, psychology, and behavioral science were invited to further review the 43-item pool, evaluating the face validity of the items, and to make the final modification suggestion. According to the suggestions of specialists, some unnecessary items were removed, such as “High-fat food will increase the risk of complications of diabetes,” and “I will feel anxious if my blood sugar level cannot be well-controlled,” and a few items were slightly reworded to improve their linguistic clarity. Finally, a total of 25 items (eight items of information, eight items of motivation, and nine items of behavioral skill) remained for subsequent analyses.

### 2.2. Study design and participants

The sample size was planned to be at least 120 in this study, with the set of α = 0.05; β = 0.2; degree of freedom (df) = 120; RMSEA = 0.05 in the null hypothesis; RMSEA = 0.08 in the test of close fit, and RMSEA = 0.01 in the test of non-close fit (37). The calculation was completed in R 4.2.2.

A descriptive cross-sectional questionnaire survey was conducted in the present study. Participants were recruited from Zhongmu, Henan province, China, and they were invited to fill out a questionnaire including demographics and DSMS by convenience sampling from November 2nd, 2021 to November 12nd, 2021. The inclusion criteria were: (1) Registered clinically diagnosed diabetic patients aged from 45 to 65 years old; (2) Fasting blood glucose level is not lower than 7.0 mmol/L or HbA1c is not lower than 6.5%; (3) Can independently finish questionnaires; (4) Can sign the informed consent form and cooperate to complete all the research contents. The exclusion criteria were: (1) Patients with serious diseases (such as malignant tumors), immunodeficiency or immunosuppressants, or those with severe neurological or mental disorders; (2) Patients who are deaf-mute, unable to move, etc. Investigators who are familiar with the local dialect were recruited and trained. Unified instructions were set for each item in the questionnaire for the investigators to ask questions, and they would fill out the questionnaire according to the answers of the participants. After the investigators and proofreaders sign at the end of each questionnaire, it is deemed that the investigation of this sample is completed. Epidata software was used for data entry and double check to ensure the accuracy of the data. In this study, 484 participants completed the questionnaires, and 469 out of them met the eligibility criteria of the study, which were employed for subsequent analysis, with an effective recovery rate of 97%. The study protocol was approved by the Ethics Committee of the Institute of Pathogen Biology, Chinese Academy of Medical Sciences (Beijing, China) (IPB-2021-09).

### 2.3. Measurement

#### 2.3.1. Demographic information

Demographic information included age, gender, educational level, marital status, annual household income in 2020, whether drank in the past year, whether smoked in the last 6 months, whether have high blood pressure, blood glucose (GLU), and glycosylated hemoglobin or glycated hemoglobin (GHB). (GLU and GHB are the current diagnostic criteria for diabetes in China) (38). GLU and GHB were measured by researchers during investigation.

#### 2.3.2. Preliminary version of diabetic self-management scale

The DSMS developed in this study was based on IMB theory, which included three dimensions: information, motivation, and behavioral skill. The information dimension consisted of eight items on knowledge of diet, physical activity, and medication of T2DM. Each item was of dichotomous response on “Yes” and “No” (“Yes” equals 1 while “No” equals 0), participants would receive 1-point for each correct response, and the higher total score of this dimension indicated a higher level of knowledge on T2DM. The Cronbach's α of this dimension was 0.630 in this study. The motivation dimension consisted of eight items and each item was 5-point Likert scaled from 1–5, and higher total scores indicated a higher level of motivation on diabetic self-management. The Cronbach's α of this dimension was 0.938 in this study. The behavioral skill dimension consisted of nine items and each item was a question that need an answer from “Yes,” “No,” or “Not clear.” Participants would receive 1-point for each correct response and get the total score after finishing all items. Higher total scores indicated a higher level of behavioral skill. The Cronbach's α of this dimension was 0.898 in this study.

### 2.4. Statistical analysis

Descriptive analysis was used to describe the demographic characteristics. Pearson correlation analysis was employed to examine the correlation among the 3 dimensions of DSMS. When assessing the psychometric properties of DSMS, the sample was randomly divided into two parts *via* a random number generator, to perform exploratory factor analysis (EFA) and confirmatory factor analysis (CFA), respectively. The final sample size of the EFA sample (sample 1) was 234 and the CFA sample (sample 2) was 235. Kaiser–Meyer–Olkin (KMO) and Bartlett's test of sphericity was used to test whether our data were suitable for factor analysis. In EFA, principal component factor analysis with varimax rotation was conducted to assess the underlying structure for the 25 items of the DSMS. Items that had a factor loading of more than 0.50 and did not load on multiple factors were obtained for further CFA (39). After EFA, three-factor CFA with oblique rotation was employed to evaluate the reliability and validity of the DSMS. Since the scales of indicators of “information” and “behavioral skill” were binary, mean and variance-adjust weight least squares (WLSMV) was used to estimate the parameters of the CFA model (40). The structural validity of the DSMS was evaluated by model fit indices, which include χ2, df, Root Mean Square Error of Approximation (RMSEA), Comparative Fit Index (CFI), Tucker-Lewis Index (TLI), and Standardized Root Mean Square Residual (SRMR). The reliability and convergent validity of the DSMS were assessed by standardized factor loadings, composite reliability (CR), and average variance extracted (AVE) (41). The discriminant validity of the DSMS was assessed by the AVE method (41). The criterion validity was assessed by the correlation among the three dimensions of DSMS and GLU and GHB (42). All the analyses were completed with SAS 9.4 and Mplus 8.3.

## 3. Results

### 3.1. Participant characteristics

A total of 469 T2DM patients were recruited in this study. 28.4% of the participants were above 60 years old, 59.9% were female, and 78.7% had high blood pressure (see Table 1).

**Table 1:** Demographic characteristics of participants (*n* = 469).

**Variables**	***N* (%)**
**Age (years)**	
45–60	336 (71.6)
>60	133 (28.4)
**Gender**	
Male	188 (40.1)
Female	281 (59.9)
**Educational level**	
Primary school or below	265 (56.5)
Above primary school	204 (43.5)
**Marital status**	
unmarried/divorced/widowed	32 (6.8)
Married	437 (93.2)
**Annual household income in 2020 (RMB)**	
≤ 30,000	294 (62.7)
>30,000	175 (37.3)
**Drink in the past year**	
No	363 (77.4)
Yes	106 (22.6)
**Smoke in the last 6 months**	
No	395 (84.2)
Yes	74 (15.8)
**High blood pressure**	
No	100 (21.3)
Yes	369 (78.7)
**GLU (mmol/L)**	9.93 ± 4.36
**GHB (%)**	10.27 ± 2.62

### 3.2. Exploratory factor analysis

The KMO measure of the 25-item original DSMS was 0.839, indicating enough items are predicted by each factor in the current study. The result of Bartlett's test of sphericity was statistically significant (χ2 = 3254.872, *P* < 0.001), suggesting that the items are correlated highly enough to provide a reasonable basis for factor analysis. In EFA, 3 major factors were expected, based on the fact that the items were designed to index 3 constructs: information, motivation, and behavioral skill. Table 2 displays the items and factor loadings for the rotated factors, with loadings < 0.50 omitted in the further analysis (39). Three items in the information dimension were deleted: “Eating too much sugar or sweet food is a cause of diabetes,” “People with diabetes cannot eat fruits and vegetables,” “If I forget to take the hypoglycemic drugs in the morning, then I can take the two drugs together at noon to make up for the morning's vacancy.”

**Table 2:** Factor loadings of 25 items in the preliminary DSMS (*n* = 234).

**Items and expected dimensions**	**Dimension**		
	**Information**	**Motivation**	**Behavioral skill**
**Information**			
Eating too much sugar or sweet food is a cause of diabetes.	**0.279**	−0.148	0.277
People with diabetes cannot eat fruits and vegetables.	**0.122**	−0.139	−0.077
Appropriate physical activities can lower my blood sugar level	**0.546**	0.198	0.046
Diabetic patients should start exercising 1/2 to 1 h after a meal	**0.703**	0.157	0.167
Diabetic patients should take sweets with them when exercising	**0.696**	−0.068	0.148
If I forget to take the hypoglycemic drugs in the morning, then I can take the two drugs together at noon to make up for the morning's vacancy	**0.411**	0.127	−0.253
All hypoglycemic agents may cause hypoglycemia	**0.563**	0.051	0.082
Paying attention to diet and strengthening physical activities are as important as taking hypoglycemic agents	**0.591**	0.294	−0.061
**Motivation**			
I attach great importance to my health	0.177	**0.616**	−0.198
It will be difficult to control my blood sugar if I do not control my diet	−0.038	**0.741**	0.119
Poor control of blood sugar can easily lead to diabetic complications (such as nephropathy, etc.)	0.168	**0.784**	−0.098
My relatives and friends around me think I should stick to the diabetes diet	0.081	**0.826**	0.161
Moderate physical activities can control my blood sugar well	0.078	**0.817**	0.128
My relatives and friends around me think I should keep moderate physical activity to control my blood sugar	0.079	**0.849**	0.184
Medication according to the doctor's advice can control my blood sugar well	0.149	**0.824**	−0.062
My relatives and friends around me think that I should stick to the doctor's advice	0.160	**0.845**	0.157
**Behavioral skill**			
When you are busy, can you still follow the dietary principles suggested by your doctor?	0.020	0.039	**0.756**
If you make up your mind, can you stick to the diabetes diet?	0.021	−0.011	**0.800**
Do you know how to eat and drink to help control blood sugar?	0.117	0.159	**0.667**
When you are busy, can you still keep exercising?	0.059	0.090	**0.820**
If you make up your mind, can you keep exercising?	0.108	0.105	**0.795**
Do you know how to exercise to help control blood sugar?	0.163	0.110	**0.755**
Can you take the medicine according to the doctor's advice during the period of taking the medicine recommended by the doctor?	−0.135	−0.043	**0.659**
If you make up your mind, can you follow the doctor's advice?	−0.075	0.048	**0.588**
Do you know how you should take medicine to control blood sugar?	0.085	0.047	**0.796**

The bold values indicate factor loadings of expected dimensions.

### 3.3. Confirmatory factor analysis

#### 3.3.1. Structural validity

The CFA model with 22 items extracted from the EFA showed χ2/df = 2.261, RMSEA = 0.073, CFI = 0.937, TLI = 0.930, and SRMR = 0.096, suggesting an acceptable model fit and structural validity of the DSMS. The model fit indices of the CFA are illustrated in Table 3.

**Table 3:** Model fit indices of the proposed model (*n* = 235).

	**χ^2^**	**df**	**χ^2^/df**	**CFI**	**TLI**	**RMSEA**	**SRMR**
Model	465.681	206	2.261	0.937	0.930	0.073	0.096

#### 3.3.2. Reliability and convergent validity

**Figure 2:**
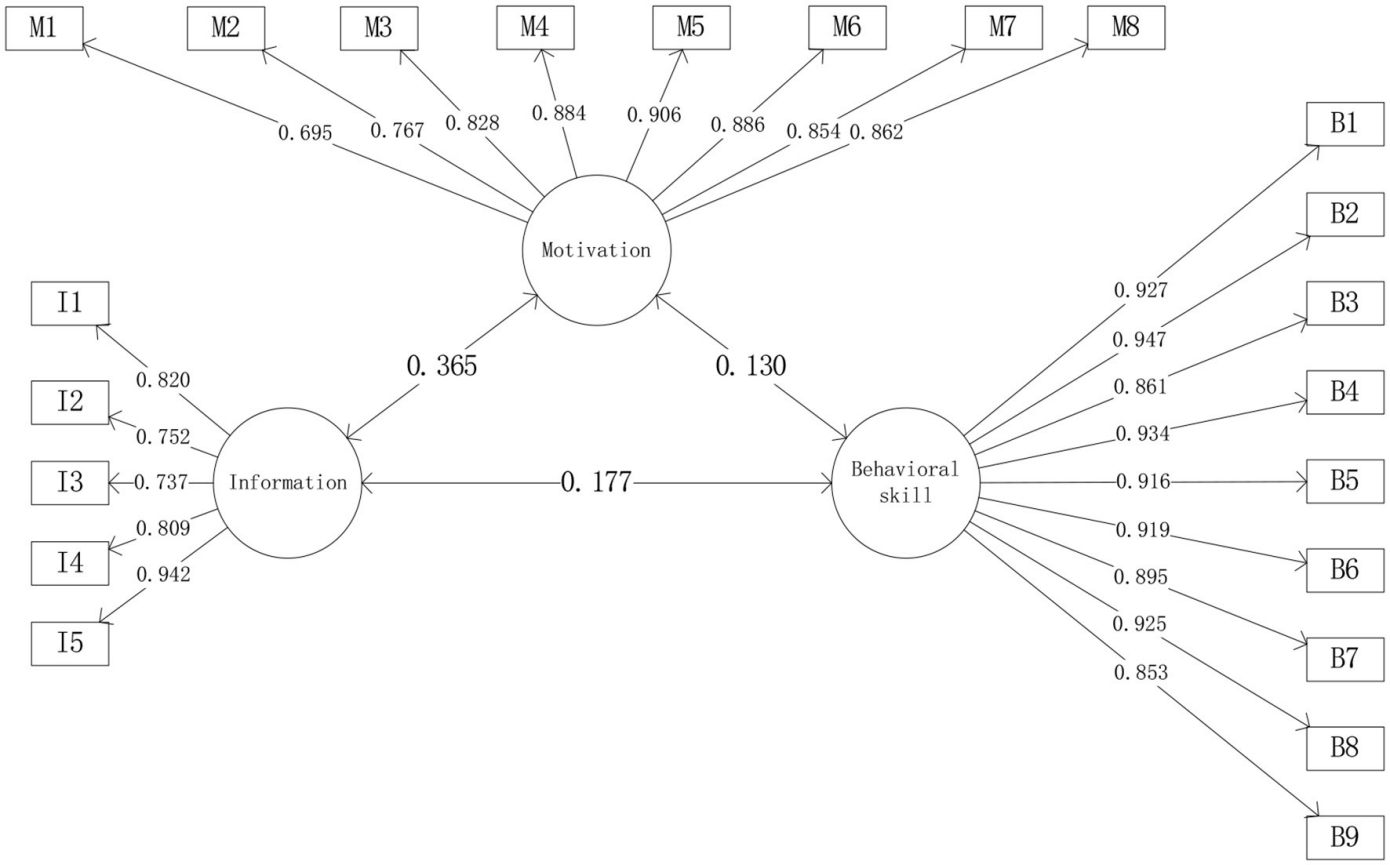
Confirmatory factor analysis of DSMS. I1–I5 are items of information; M1–M8 are items of motivation; B1–B9 are items of behavioral skill.

The standardized factor loadings of 22-item DSMS were all above 0.6 and statistically significant. The values of CRs for the three dimensions were all above 0.9. The AVEs of the dimensions were above 0.6, suggesting good reliability and convergent validity of the DSMS (see Table 4, Figure 2).

**Table 4:** Reliability and convergent validity of 22-item DSMS (*n* = 235).

**Construct**	**Indicator**	**STD**	**S.E**.	**Z**	**P**	**CR**	**AVE**
Information	Appropriate physical activities can lower my blood sugar level	0.820	0.067	12.194	< 0.001	0.908	0.664
	Diabetic patients should start exercising half an hour to 1 h after a meal	0.752	0.061	12.406	< 0.001		
	Diabetic patients should take sweets with them when exercising	0.737	0.064	11.527	< 0.001		
	All hypoglycemic agents may cause hypoglycemia	0.809	0.063	12.789	< 0.001		
	Paying attention to diet and strengthening physical activities are as important as taking hypoglycemic agents	0.942	0.055	17.029	< 0.001		
Motivation	I attach great importance to my health	0.695	0.020	34.477	< 0.001	0.949	0.702
	It will be difficult to control my blood sugar if I do not control my diet	0.767	0.017	46.418	< 0.001		
	Poor control of blood sugar can easily lead to diabetic complications (such as nephropathy, etc.)	0.828	0.013	66.076	< 0.001		
	My relatives and friends around me think I should stick to the diabetes diet	0.884	0.010	84.458	< 0.001		
	Moderate physical activities can control my blood sugar well	0.906	0.007	127.555	< 0.001		
	My relatives and friends around me think I should keep moderate physical activity to control my blood sugar	0.886	0.010	85.441	< 0.001		
	Medication according to the doctor's advice can control my blood sugar well	0.854	0.009	96.811	< 0.001		
	My relatives and friends around me think that I should stick to the doctor's advice	0.862	0.012	70.752	< 0.001		
Behavioral skill	When you are busy, can you still follow the dietary principles suggested by your doctor?	0.927	0.022	42.545	< 0.001	0.977	0.826
	If you make up your mind, can you stick to the diabetes diet?	0.947	0.025	38.425	< 0.001		
	Do you know how to eat and drink to help control blood sugar?	0.861	0.030	28.944	< 0.001		
	When you are busy, can you still keep exercising?	0.934	0.021	45.399	< 0.001		
	If you make up your mind, can you keep exercising?	0.916	0.023	40.319	< 0.001		
	Do you know how to exercise to help control blood sugar?	0.919	0.023	39.335	< 0.001		
	Can you take the medicine according to the doctor's advice during the period of taking the medicine recommended by the doctor?	0.895	0.030	29.867	< 0.001		
	If you make up your mind, can you follow the doctor's advice?	0.925	0.028	33.400	< 0.001		
	Do you know how you should take medicine to control blood sugar?	0.853	0.031	27.200	< 0.001		

STD, Standardized factor loading; S.E., Standard error; CR, Composite reliability; AVE, Average variance extracted.

#### 3.3.3. Discriminant and criterion validity

To evaluate discriminant and criterion validity, the correlations of 3 dimensions were examined. When the square root of each factor's AVE is greater than the absolute value of the correlation between this dimension and the other two dimensions, the model demonstrates discriminant validity. As is shown in Table 5, the diagonal elements in the correlation matrix of DSMS factors were the square root of AVE. All the diagonal elements were greater than corresponding off-diagonal elements, indicating the DSMS showed good discriminant validity. Additionally, criterion validity was evaluated by the correlation between 3 dimensions of DSMS and GLU, and GHB. As shown in Table 5, three dimensions of DSMS were all negatively associated with GLU and GHB, except the correlation between behavioral skill and GHB, but this correlation was not statistically significant. Additionally, despite the non-significant correlation between motivation and GLU, the correlation between motivation and GHB were statistically significant, indicating that DSMS developed in this study had good criterion validity.

**Table 5:** Discriminant and criterion validity of 22-item DSMS.

	**Information**	**Motivation**	**Behavioral skill**
Information	**0.815**		
Motivation	0.365^*^	**0.838**	
Behavioral skill	0.177^*^	0.130^*^	**0.909**
GLU	−0.196^*^	−0.068	−0.148^*^
GHB	−0.091^*^	−0.171^*^	0.015

^*^P < 0.05. The diagonal bold values indicate the correlation matrix of DSMS factors were the square root of AVE.

### 3.4. Characteristics of dimensions in DSMS

The average scores of information, motivation, and behavioral skill for all participants were (2.697 ± 1.607), (4.073 ± 0.517), and (6.058 ± 3.033) (see Table 6).

**Table 6:** Average scores of 3 dimensions in 22-item DSMS.

	**Min**	**Max**	**Mean**	**SD**
Information	0.00	5.00	2.6972	1.60728
Motivation	1.00	5.00	4.0728	0.51720
Behavioral skill	0.00	9.00	6.0576	3.03310

Min, minimum; Max, maximum; SD, standard deviation.

## 4. Discussion

T2DM is a chronic disease requiring patients' lifelong self-management to avoid the occurrence of complications and to ensure the achievement of the best clinical outcomes (43, 44). Recognizing the weak links and misconceptions of self-management of T2DM patients in China is of positive significance for the health authorities and medical care workers to implement targeted intervention among this population. Therefore, it is necessary to develop a scale to comprehensively assess the self-management status of T2DM patients. The primary aim of this study was to develop a scale to evaluate the self-management ability, including diet, physical activity, and medication, of middle-aged and elderly T2DM patients in China based on IMB theory. The final DSMS is a 22-item scale with a systematic yet simple 3-factor structure encapsulating information, motivation, and behavioral skill, which is consistent with the established theoretical perspectives of IMB (34). The standardized factor loadings of 22 items in CFA were all above the recommended value of 0.5 and statistically significant, indicating good communalities of items (39). The value of Cronbach's α and CR for the three dimensions were all above 0.7, indicating good reliability and convergent validity of the CFA model (41, 45). In addition, the discriminant validity of the CFA model was acceptable according to the comparison between correlation coefficients and AVEs. These findings provide reasonable evidence that DSMS has satisfied psychometric properties to meet the requirement for a self-report measure of self-management ability among Chinese middle-aged and elderly T2DM patients.

Blood sugar is an important indicator to evaluate the self-management of T2DM patients (46, 47). The three dimensions of DSMS were all negatively associated with hyperglycemia markers, such as higher levels of GHB and GLU. The first dimension is information, which is the precondition for healthy behavior in the IMB model. Researchers have pointed out that information can directly affect diabetes patients' self-management behavior, and a high level of diabetes-related knowledge is beneficial for patients' glycemic control (34, 48). In addition, sufficient diabetes-related information can improve drug compliance and regular glycemic monitoring of T2DM patients, which will also help to avoid the deterioration of the disease (32, 49). The second dimension is motivation, including personal motivation and social motivation, which are independent and direct predictors of T2DM self-management behavior (50). Researches indicated that positive motivation can promote physical activity and a healthy diet in T2DM patients (49, 51). However, the relationship between motivation and blood glucose monitoring and drug compliance has not been identified, since these behaviors may be more strictly limited by information such as doctor's advice, while the diet and physical activity are more flexible and can be easily adjusted according to the patient's motivation (52). The last part of the IMB model is behavior skills. Behavior skills are composed of personal objective skills and self-efficacy, which are also positively associated with diabetes self-management behavior (53). Previous studies have shown that information and motivation are positively associated with behavioral skills, and behavioral skills are also positively associated with T2DM self-care behaviors (54). Although information and motivation are essential for self-management behaviors in T2DM patients, it might be difficult to adopt correct healthy behaviors without solid practical skills (50). In addition, HIV-related intervention studies suggested that behavioral skills mediate the relationship among information, motivation, and health behaviors (55, 56). Therefore, DSMS developed in this study might give a direct evaluation of the health outcome of T2DM self-management. Based on the DSMS, health authorities and medical care workers can understand the factors of poor self-management behavior of T2DM patients and then take targeted health education and intervention.

In this study, all the items in the dimensions of motivation and behavioral skill of the preliminary DSMS have been completed and retained, indicating that the items of these two dimensions could well-assess the self-management of T2DM patients. However, in EFA, we found three redundant items and deleted them in the “Information” dimension from the original version of DSMS due to their low factor loadings and poor interpretability to the whole DSMS. Since the launch of the new round of health system reform in China in 2009, community-based diabetes management and care has become one of the key contents of the country's basic public health services, including regular blood glucose testing, the guidance of medication, diet control, and physical activity, which has helped to improve the knowledge and self-care awareness level of T2DM patients (57–59). In this case, the deleted three items in “Information” dimension were more like common sense for T2DM patients, which might be too simple for them, thus demonstrating low consistency with other items.

This study has several strengths. First, the 22-item scale is relatively appropriate for the middle-aged and elderly and is comparable in length to other widely used measures, such as DSCS which has 26 items (26). Second, researchers in this study had background in diabetic epidemiology and were familiar with psychology and behavioral science, which could ensure the face and content validity of the scale. Third, during the scale-development process, we employed the IMB model as our theoretical framework and considered the items from aspects of diet, physical activity, and medication, which provided a systematic and comprehensive assessment for the T2DM. Moreover, all 469 T2DM patients fully completed the questionnaires, indicating the acceptability of the scale, and the sample size was large enough for the psychometric testing.

This study also has several limitations. First, this study is limited by the convenience sampling method and the fact that it consisted only of middle-aged and elderly T2DM patients in Zhongmu, Henan, China. Further studies including T2DM patients of different ages and regions are necessary to examine the validity of DSMS in the Chinese context. Second, the CR values of the three subscales were all above 0.9, despite displaying good reliability of the scale, it suggested that further scale reduction might be applicable, however, further deletion may reduce the face and content validity of the scale. Third, this study may also be limited by the fact that the DSMS is a self-report instrument. Participants may give a subjective or socially appropriate answer that does not reflect their true thoughts. Further comprehensive investigation with the additional use of qualitative methods may be valuable.

## 5. Conclusion

Currently, no instrument can be used to systematically assess the self-management readiness of middle-aged and elderly T2DM patients in China. The 22-item DSMS developed in our study is an important step toward closing this gap, and can be used to make comprehensive assessment of diabetic self-management regarding diet, physical activity, and medication, based on IMB theory. The DSMS is validated with good reliability and validity, with moderate length and understandable content for middle-aged and elderly T2DM patients in China. Thus, the DSMS can be applied in China to identify levels of self-management among middle-aged and elderly T2DM patients in China.

## Data availability statement

The raw data supporting the conclusions of this article will be made available by the authors, without undue reservation.

## Ethics statement

The studies involving human participants were reviewed and approved by the Ethics Committee of the Institute of Pathogen Biology, Chinese Academy of Medical Sciences (Beijing, China) (IPB-2021-09). The patients/participants provided their written informed consent to participate in this study.

## Author contributions

XS, ZD, SJ, and XL prepared the first draft. XS provided overall guidance and managed the overall project. ZD, SJ, XL, HZ, YW, HW, WX, YH, JF, XC, and LG were responsible for the questionnaire survey, intervention implementation, and data analysis. All authors contributed to the article and approved the submitted version.
